# Supplementary material for: Sequence and parent-of-origin dependent m6A contribute to allele-specific gene expression
Source: EMBO J. 2025 Jun 3;44(14):4120–49. doi: 10.1038/s44318-025-00476-3 (PMC12264043; doi:10.1038/s44318-025-00476-3)
Supplement: Supplementary file 11 — Expanded View Figures [file 44318_2025_476_MOESM11_ESM.pdf]

Expanded View Figures

Figure EV1. Allele-specific analysis of m^{6}A methylation profiles.

(**A**) Workflow for upstream data analysis to identify ASm^{6}A sites. (**B**–**D**, **F**) Metagene profiles showing the distributions of m^{6}A peak summits (**B**), GLORI-detected m^{6}A sites (**C**), high-confidence m^{6}A sites (**D**) and allelically detectable m^{6}A sites (**F**). Motif analysis of high-confidence m^{6}A sites is shown in (**D**). (**E**) Upset plot illustrating intersections of high-confidence m^{6}A sites across all samples. The top 30 intersections, ranked by size, are displayed. The deep blue highlights the intersection of all samples. The pie chart shows the distribution of m^{6}A sites identified in all 14 samples, 3–13 samples, and fewer than 3 samples. (**G**) PCA of allele-specific m^{6}A levels in 12 cerebellum sub-samples. Each dot represents an allele-specific sub-sample, with shape denoting sex (circle for female, triangle for male) and size denoting age (small for P0, large for P7). Dot color indicates genotype, with outline color denoting parent-of-origin and fill color denoting strain. The first two PCs are shown. Further components are shown in (**H**). PC, principal component. (**H**) Scree plots presenting the percentage of explained variances for identified components in PCA analyses. Color scheme for samples depicted in (**D**–**F**) is illustrated in the legend of (**B**).

© The Author(s)

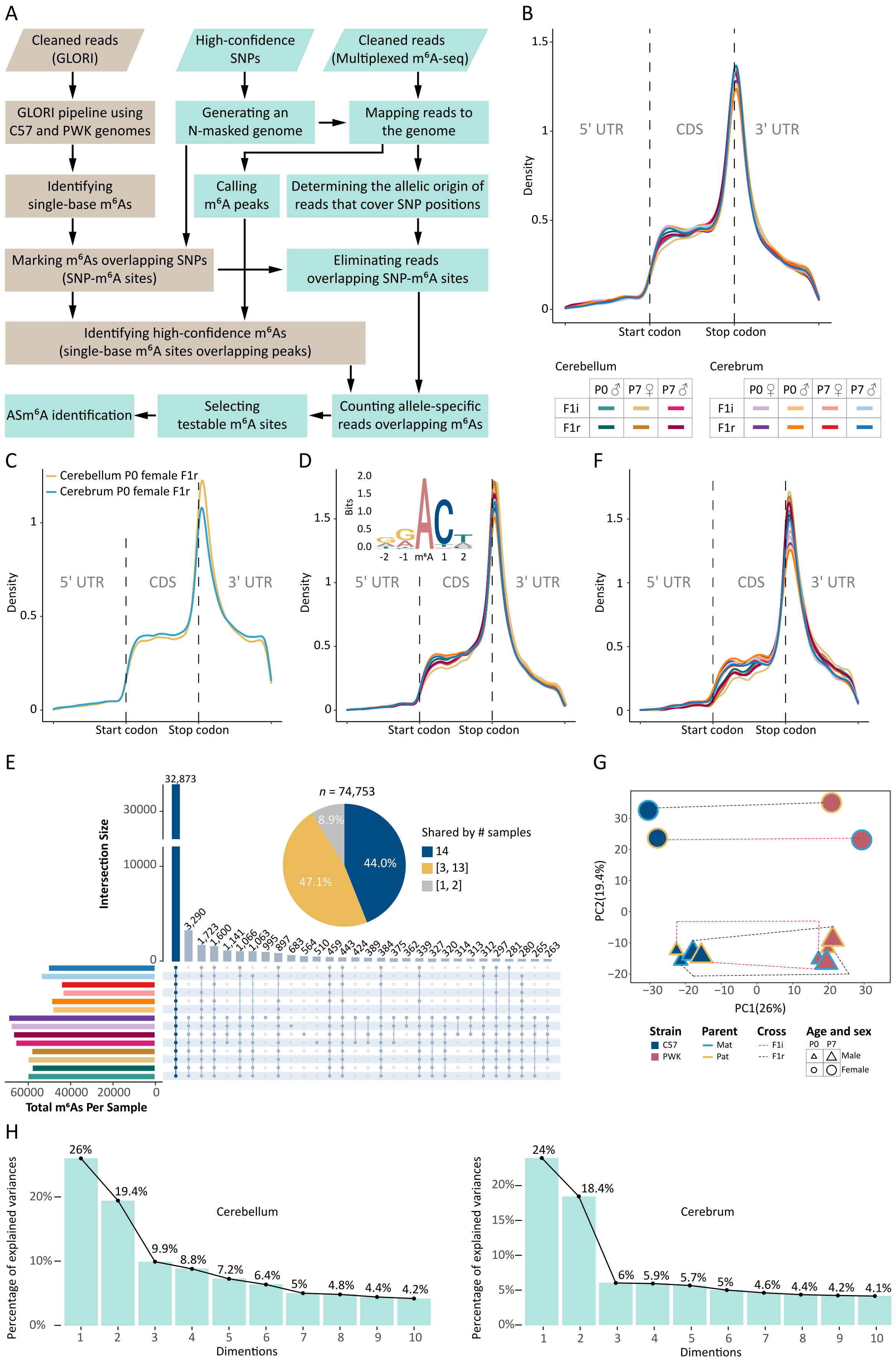
A
Cleaned reads (GLORI)
High-confidence SNPs
Cleaned reads (Multiplexed m6A-seq)
GLORI pipeline using C57 and PWK genomes
Generating an N-masked genome
Mapping reads to the genome
Identifying single-base m6As
Calling m6A peaks
Determining the allelic origin of reads that cover SNP positions
Marking m6As overlapping SNPs (SNP-m6A sites)
Eliminating reads overlapping SNP-m6A sites
Identifying high-confidence m6As (single-base m6A sites overlapping peaks)
ASm6A identification
Selecting testable m6A sites
Counting allele-specific reads overlapping m6As
B
5' UTR
CDS
3' UTR
Density
Start codon
Stop codon
Cerebellum
Cerebrum
P0 ♂
P7 ♀
P7 ♂
P0 ♀
F1i
F1r
C
Cerebellum P0 female F1r
Cerebrum P0 female F1r
D
Bits
m6A
F
E
Intersection Size
n = 74,753
Shared by # samples
14
[3, 13]
[1, 2]
44.0%
47.1%
8.9%
Total m6As Per Sample
G
PC2(19.4%)
PC1(26%)
Strain
C57
PWK
Parent
Mat
Pat
Cross
F1i
F1r
Age and sex
P0
P7
Male
Female
H
Percentage of explained variances
Cerebellum
Cerebrum
Dimentions

 © The Author(s)

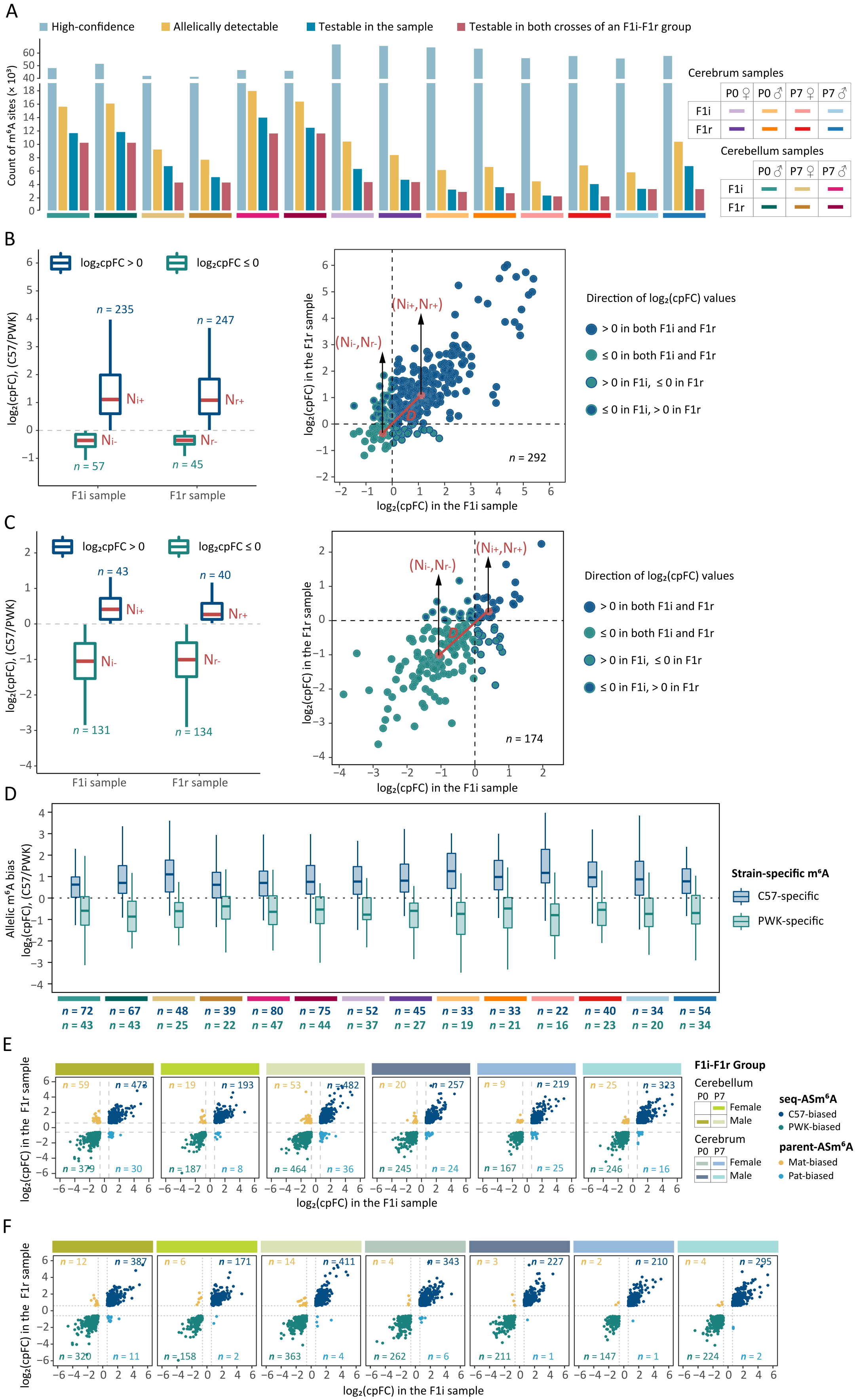
A
High-confidence
Allelically detectable
Testable in the sample
Testable in both crosses of an F1i-F1r group
Count of m6A sites (× 10³)
Cerebrum samples
Cerebellum samples
F1i
F1r
B
log₂cpFC > 0
log₂cpFC ≤ 0
log₂(cpFC), (C57/PWK)
F1i sample
F1r sample
log₂(cpFC) in the F1r sample
log₂(cpFC) in the F1i sample
n = 292
Direction of log₂(cpFC) values
> 0 in both F1i and F1r
≤ 0 in both F1i and F1r
> 0 in F1i, ≤ 0 in F1r
≤ 0 in F1i, > 0 in F1r
C
n = 174
D
Allelic m6A bias
log₂(cpFC), (C57/PWK)
Strain-specific m6A
C57-specific
PWK-specific
E
log₂(cpFC) in the F1r sample
log₂(cpFC) in the F1i sample
F1i-F1r Group
Cerebellum
Cerebrum
Female
Male
seq-ASm6A
C57-biased
PWK-biased
parent-ASm6A
Mat-biased
Pat-biased
F

Figure EV2. Identification of candidate ASm^{6}A sites.

(**A**) Bar plots displaying counts of m^{6}A sites categorized into four groups (see "Methods"). ASm^{6}A identification within each F1i-F1r group utilized sites testable in both crosses. (**B**, **C**) Evaluation of the fluctuation in $\log_2(cpFC)$ values of positive controls. Positive controls encompass both C57-specific (**B**) and PWK-specific (**C**) m^{6}A sites. Box plots depict the distribution of positive and negative $\log_2(cpFC)$ values within F1i and F1r samples, resulting in four datasets whose medians are used to evaluate the fluctuation (see "Methods"). N_{i+} and N_{r+} denote the medians of positive $\log_2(cpFC)$ values in F1i and in F1r samples, respectively. Similarly, N_{i-} and N_{r-} denote the medians of negative $\log_2(cpFC)$ values in F1i and in F1r samples, respectively. D represents the overall fluctuation level of $\log_2(cpFC)$ values in the F1i-F1r group, which was assessed using Euclidean distance (see "Methods"). (**D**) Box plot illustrating allelic m^{6}A difference for positive controls across all m^{6}A-seq samples. Color scheme for the samples is identical to that of (**A**). (**E**) Four-quadrant scatter plots illustrating ASm^{6}A distribution across six F1i-F1r groups. (**F**) Four-quadrant scatter plots showing highly reproducible ASm^{6}A sites. These sites were chosen from ASm^{6}A sites within each F1i-F1r group based on two criteria: each ASm^{6}A site must be detectable in ≥ 4 samples, and its group-level allelic bias must align directionally with the tissue-level allelic bias (see "Methods"). The color scheme is consistent with that of (**E**). (**B**–**D**) The top, middle, and bottom lines of the box represent the upper quartile (Q3), median, and lower quartile (Q1), respectively. The upper whisker extends to the maximum value provided it is not larger than $(Q3 + 1.5\times IQR)$ (where $IQR = Q3 - Q1$), while the lower whisker extends to the minimum value provided it is not smaller than $(Q1 - 1.5\times IQR)$. Data points beyond the whiskers are considered outliers and are not displayed. Source data are available online for this figure.

 © The Author(s)

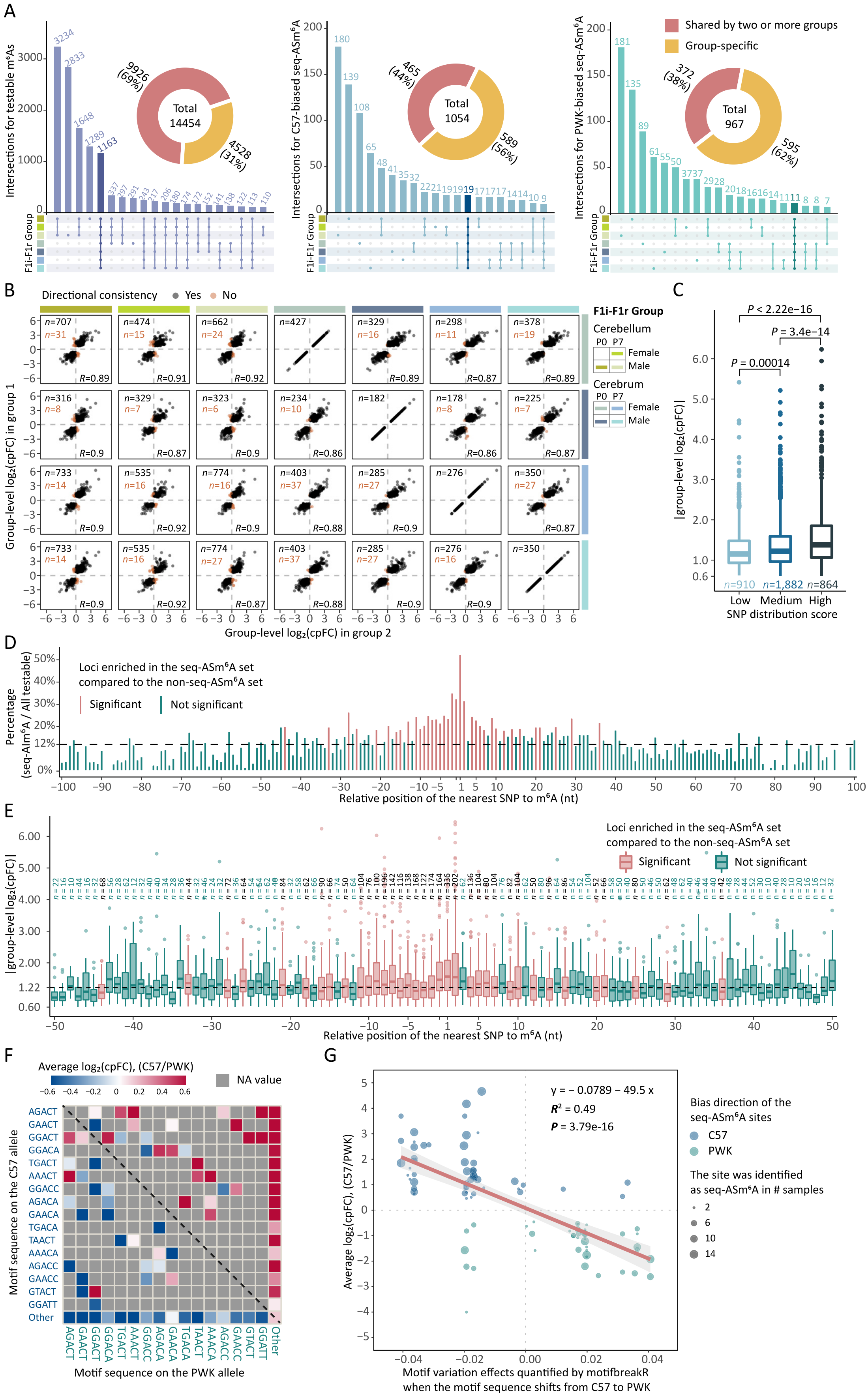
A
Intersections for testable m6As
F1i-F1r Group
Total
14454
9926
(69%)
4528
(31%)
Intersections for C57-biased seq-ASm6A
Total
1054
465
(44%)
589
(56%)
Intersections for PWK-biased seq-ASm6A
Shared by two or more groups
Group-specific
Total
967
372
(38%)
595
(62%)
B
Directional consistency Yes No
F1i-F1r Group
Cerebellum
P0 P7
Female
Male
Cerebrum
P0 P7
Female
Male
Group-level log2(cpFC) in group 1
Group-level log2(cpFC) in group 2
C
P < 2.22e−16
P = 3.4e−14
P = 0.00014
|group-level log2(cpFC)|
n=910 n=1,882 n=864
Low Medium High
SNP distribution score
D
Loci enriched in the seq-ASm6A set
compared to the non-seq-ASm6A set
Significant
Not significant
Percentage
(seq-AIm6A / All testable)
Relative position of the nearest SNP to m6A (nt)
E
Loci enriched in the seq-ASm6A set
compared to the non-seq-ASm6A set
Significant
Not significant
|group-level log2(cpFC)|
Relative position of the nearest SNP to m6A (nt)
F
Average log2(cpFC), (C57/PWK)
NA value
Motif sequence on the C57 allele
Motif sequence on the PWK allele
G
y = − 0.0789 − 49.5 x
R2 = 0.49
P = 3.79e−16
Average log2(cpFC), (C57/PWK)
Motif variation effects quantified by motifbreakR
when the motif sequence shifts from C57 to PWK
Bias direction of the
seq-ASm6A sites
C57
PWK
The site was identified
as seq-ASm6A in # samples

Figure EV3. *Cis*-regulatory effects on allelic m^{6}A levels.

(**A**) Upset plots illustrating intersections of testable m^{6}A sites (left), C57-biased seq-ASm^{6}A (middle) and PWK-biased seq-ASm^{6}A (right) across all F1i-F1r groups. The top 20 intersections, ranked by size, are displayed. The deeper color within each plot highlights the intersection of all groups. Donut charts adjacent to each plot show the proportions of group-specific and group-shared sites. (**B**) Correlation analysis of allelic bias in seq-ASm^{6}A methylation across F1i-F1r groups. Each scatterplot depicts the intersection between seq-ASm^{6}A sites in a cerebrum group (Group 1) and allelically detectable m^{6}A sites in another group (Group 2). Pearson's *R* and the count of m^{6}A sites are annotated. (**C**) Box plot showing the allelic imbalance levels of seq-ASm^{6}A sites categorized by SNP distribution scores (see "Methods"). Statistical analysis utilized the two-sided Wilcoxon rank-sum test. (**D**) Enrichment analysis of SNP positions in the flanking 100 nt region of seq-ASm^{6}A sites (see "Methods"). Each bar indicates the ratio of seq-ASm^{6}A sites to all testable m^{6}A sites with their nearest SNP at the position. The dashed line indicates the average ratio. Statistical analysis employed a one-sided Binomial test (*$P < 0.05$, **$P < 0.01$, ***$P < 0.0001$). (**E**) Box plots illustrating the distribution of allelic m^{6}A imbalance levels for seq-ASm^{6}As with the nearest SNP at specific positions. For each position, the count (*n*) of seq-ASm^{6}A sites with their nearest SNP located at that position is labeled. (**F**) Heatmap depicting allelic m^{6}A differences among motif pairs in the cerebrum (see "Methods"). Motifs are ranked by occurrence frequency within all high-confidence m^{6}A sites in the cerebrum. For each motif pair, color represents the average $\log_2(cpFC)$ value of m^{6}A sites with corresponding motif variations. Blue and red indicate higher m^{6}A levels on the PWK and C57 alleles, respectively. Gray represents NA values. (**G**) Linear regression analysis of motif variation effects and allelic m^{6}A differences in cerebral seq-ASm^{6}As. The variation effects were determined using motifbreakR (see "Methods"). Shaded areas represent 95% confidence intervals of the regression line. Statistical significance was assessed using the Student's *t* test. (**D**, **E**) SNP positions are labeled with respect to the transcript strand. (**C**, **E**) The top, middle, and bottom lines of the box represent the upper quartile (Q3), median, and lower quartile (Q1), respectively. The upper whisker extends to the maximum value provided it is not larger than ($Q3 + 1.5\times IQR$) (where $IQR = Q3 - Q1$), while the lower whisker extends to the minimum value provided it is not smaller than ($Q1 - 1.5\times IQR$). Data points beyond the whiskers are considered outliers and are plotted individually.

 © The Author(s)

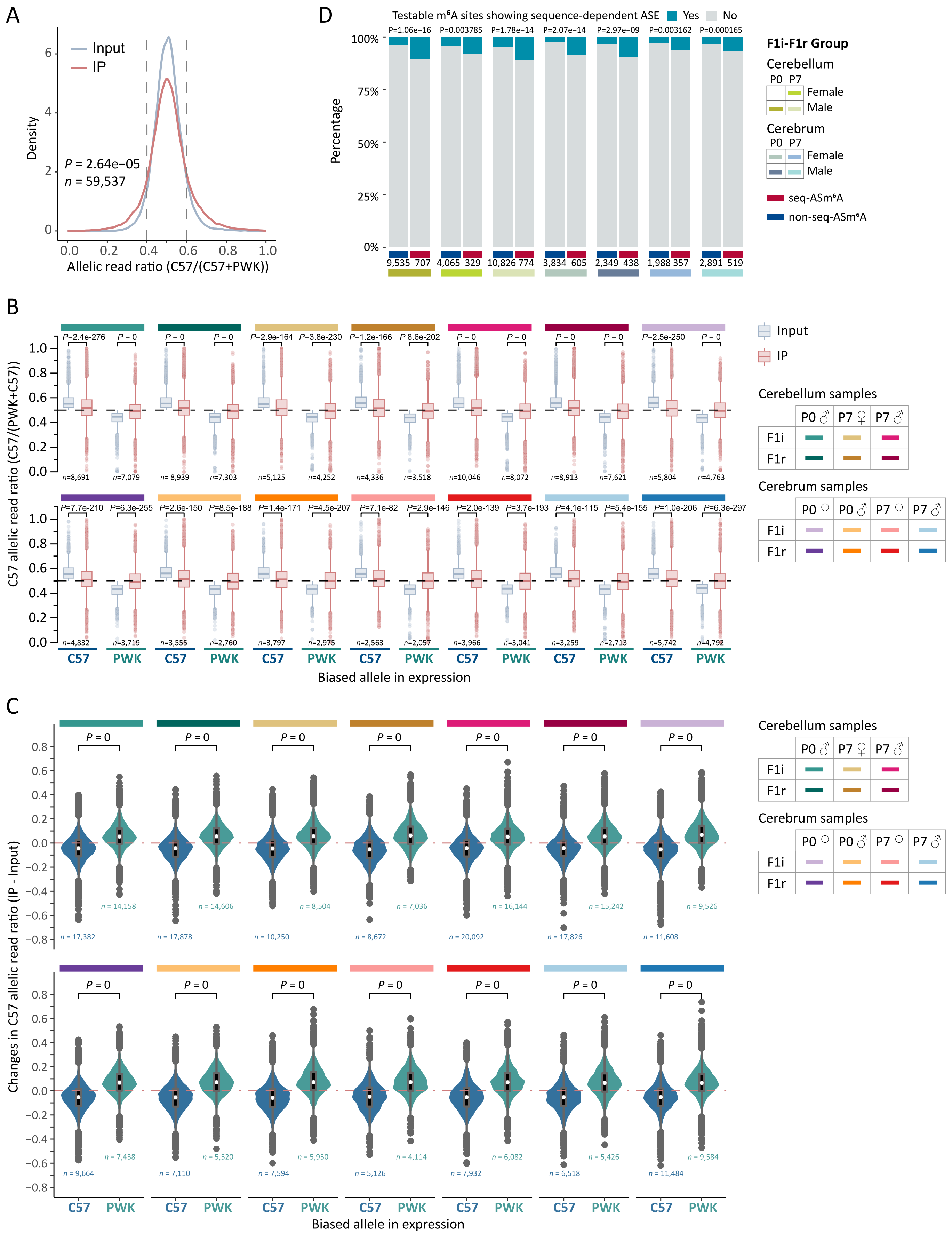
A
Input
IP
Density
P = 2.64e−05
n = 59,537
Allelic read ratio (C57/(C57+PWK))
D
Testable m6A sites showing sequence-dependent ASE
Yes
No
P=1.06e−16
P=0.003785
P=1.78e−14
P=2.07e−14
P=2.97e−09
P=0.003162
P=0.000165
Percentage
9,535 707
4,065 329
10,826 774
3,834 605
2,349 438
1,988 357
2,891 519
F1i-F1r Group
Cerebellum
P0 P7
Female
Male
Cerebrum
P0 P7
Female
Male
seq-ASm6A
non-seq-ASm6A
B
Input
IP
Cerebellum samples
P0 ♂ P7 ♀ P7 ♂
F1i
F1r
Cerebrum samples
P0 ♀ P0 ♂ P7 ♀ P7 ♂
F1i
F1r
C57 allelic read ratio (C57/(PWK+C57))
P=2.4e-276 P = 0
P = 0 P = 0
P=2.9e-164 P=3.8e-230
P=1.2e-166 P 8.6e-202
P = 0 P = 0
P = 0 P = 0
P=2.5e-250 P = 0
n=8,691 n=7,079 n= 8,939 n=7,303 n=5,125 n=4,252 n=4,336 n=3,518 n=10,046 n=8,072 n=8,913 n=7,621 n=5,804 n=4,763
P=7.7e-210 P=6.3e-255
P=2.6e-150 P=8.5e-188
P=1.4e-171 P=4.5e-207
P=7.1e-82 P=2.9e-146
P=2.0e-139 P=3.7e-193
P=4.1e-115 P=5.4e-155
P=1.0e-206 P=6.3e-297
n=4,832 n=3,719 n=3,555 n=2,760 n=3,797 n=2,975 n=2,563 n=2,057 n=3,966 n=3,041 n=3,259 n=2,713 n=5,742 n=4,792
C57 PWK
Biased allele in expression
C
Cerebellum samples
P0 ♂ P7 ♀ P7 ♂
F1i
F1r
Cerebrum samples
P0 ♀ P0 ♂ P7 ♀ P7 ♂
F1i
F1r
Changes in C57 allelic read ratio (IP - Input)
P = 0
n = 17,382 n = 14,158 n = 17,878 n = 14,606 n = 10,250 n = 8,504 n = 8,672 n = 7,036 n = 20,092 n = 16,144 n = 17,826 n = 15,242 n = 11,608 n = 9,526
n = 9,664 n = 7,438 n = 7,110 n = 5,520 n = 7,594 n = 5,950 n = 5,126 n = 4,114 n = 7,932 n = 6,082 n = 6,518 n = 5,426 n = 11,484 n = 9,584
C57 PWK
Biased allele in expression

© The Author(s)

Figure EV4. Opposing allelic preferences between sequence-dependent m^{6}A methylation and expression.

(**A**) Density plot illustrating allelic read ratio distributions in untreated input and IP samples for all allelically detectable m^{6}A sites. Statistical significance was assessed using the two-tailed paired Student's *t* test. (**B**) Box plots comparing C57 allelic read ratios between untreated input and IP samples. Statistical analysis utilized the two-sided paired Wilcoxon rank-sum test. $***P < 0.0001$. (**C**) Distributions of differences in C57 allelic read ratios between untreated input and IP samples. White circle indicates median, and violin-shaped areas depict kernel density estimates of data distribution. Statistical analysis employed the two-sided Wilcoxon rank-sum test. (**D**) Stacked bar charts showing the proportions of testable m^{6}A sites with ASE and without ASE. Comparison was conducted between seq-ASm^{6}A and non-seq-ASm^{6}A sites using the two-sided Pearson's chi-squared test. The bottom numbers indicate the number of m^{6}A sites. (**B**, **C**) The top, middle, and bottom lines of the box represent the upper quartile (Q3), median, and lower quartile (Q1), respectively. The upper whisker extends to the maximum value provided it is not larger than $(Q3 + 1.5\times IQR)$ (where $IQR = Q3 - Q1$), while the lower whisker extends to the minimum value provided it is not smaller than $(Q1 - 1.5\times IQR)$. Data points beyond the whiskers are considered outliers and are plotted individually. The count (*n*) of m^{6}A sites for each plot is labeled in the figure.

 © The Author(s)

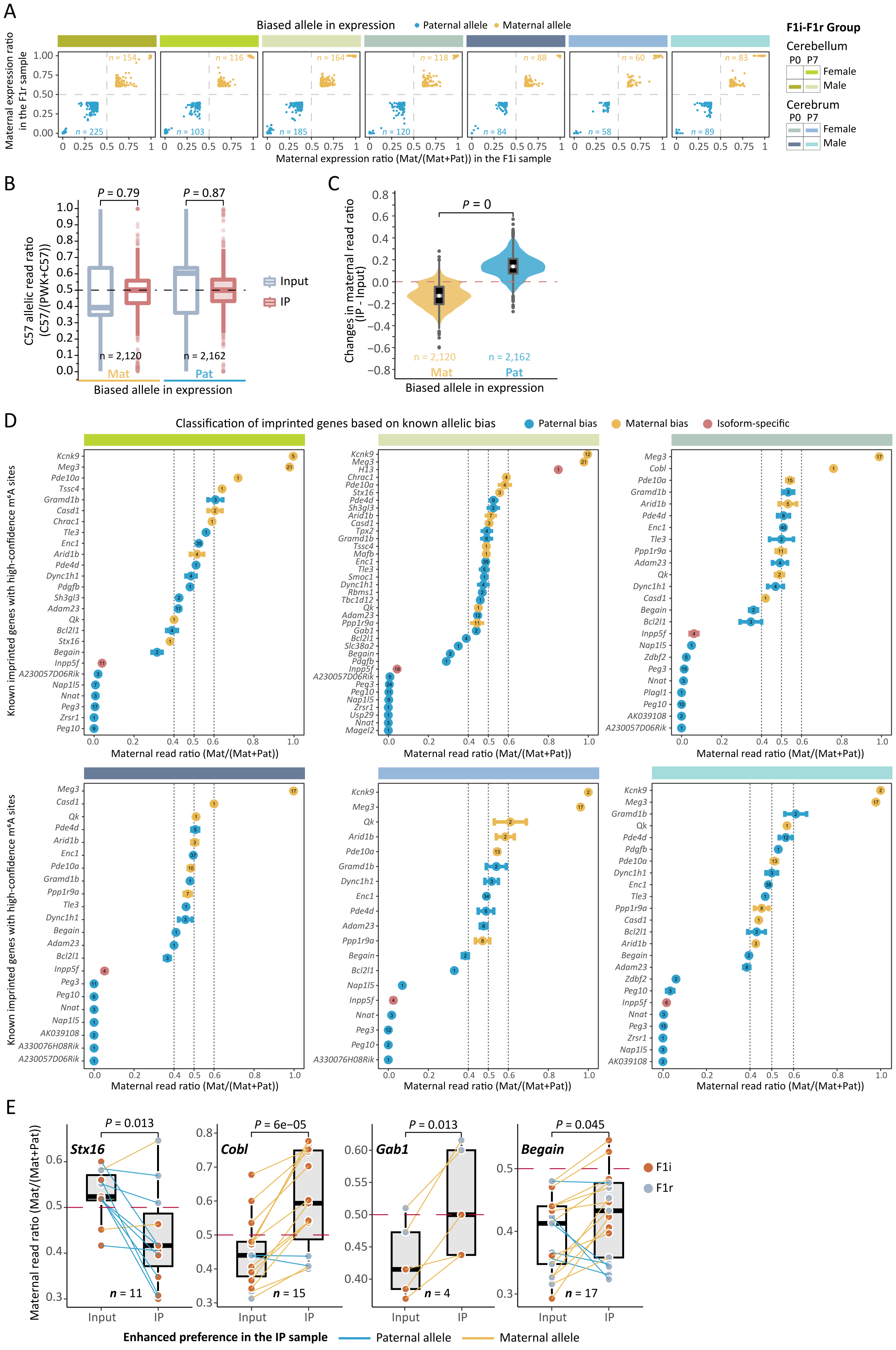
A
Biased allele in expression
Paternal allele
Maternal allele
F1i-F1r Group
Cerebellum
P0 P7
Female
Male
Cerebrum
P0 P7
Female
Male
Maternal expression ratio in the F1r sample
Maternal expression ratio (Mat/(Mat+Pat)) in the F1i sample
n = 154
n = 225
n = 116
n = 103
n = 164
n = 185
n = 118
n = 120
n = 88
n = 84
n = 60
n = 58
n = 83
n = 89
B
P = 0.79
P = 0.87
C57 allelic read ratio (C57/(PWK+C57))
Input
IP
n = 2,120
n = 2,162
Mat
Pat
Biased allele in expression
C
P = 0
Changes in maternal read ratio (IP - Input)
n = 2,120
n = 2,162
Mat
Pat
Biased allele in expression
D
Classification of imprinted genes based on known allelic bias
Paternal bias
Maternal bias
Isoform-specific
Known imprinted genes with high-confidence m6A sites
Maternal read ratio (Mat/(Mat+Pat))
E
P = 0.013
Stx16
n = 11
P = 6e−05
Cobl
n = 15
P = 0.013
Gab1
n = 4
P = 0.045
Begain
n = 17
F1i
F1r
Maternal read ratio (Mat/(Mat+Pat))
Input
IP
Enhanced preference in the IP sample
Paternal allele
Maternal allele

Figure EV5. Parental effects on m^6A methylation and expression.

(**A**) Scatter plots showing m^6A sites exhibiting parent-of-origin-dependent ASE in each F1i-F1r group. These sites were identified based on maternal read ratio in untreated input samples (see "Methods"). (**B**) Box plots comparing C57 allelic read ratios between untreated input and IP samples, with m^6A sites exhibiting maternal ($n = 2120$) and paternal ($n = 2162$) allelic expression biases shown separately. Statistical analysis utilized the two-sided paired Wilcoxon rank-sum test. (**C**) Distributions of differences in maternal allelic read ratios between untreated input and IP samples. Statistical analysis employed the two-sided Wilcoxon rank-sum test. White circle indicates median, and violin-shaped areas depict kernel density estimates of data distribution. (**D**) Imprinted genes harboring high-confidence m^6A sites in each F1i-F1r group. Each point represents a gene, color-coded by its reported imprinted category and labeled with the number of m^6A sites. Maternal expression ratio is shown as mean ± standard error across all m^6A sites within each gene. The genes are ranked by maternal read ratio. The dotted lines represent the cutoffs for identifying m^6A sites showing parent-of-origin-dependent ASE (see "Methods"). Color scheme for groups is provided in (**A**). (**E**) Box plots illustrating differences in maternal allelic read ratios between untreated input and IP samples for allelically detectable m^6A sites within known imprinted genes. Four representative genes are shown, with the count (n) of m^6A sites labeled in the plot. Sites are labeled by cross (F1i or F1r) to distinguish sequence- and parent-of-origin-dependent effects. Statistical significance was assessed using the two-tailed paired Student's t test. (**B**, **C**, **E**) The top, middle, and bottom lines of the box represent the upper quartile (Q3), median, and lower quartile (Q1), respectively. The upper whisker extends to the maximum value provided it is not larger than ($Q3 + 1.5\times IQR$) (where $IQR = Q3 - Q1$), while the lower whisker extends to the minimum value provided it is not smaller than ($Q1 - 1.5\times IQR$). Data points beyond the whiskers are considered outliers and are plotted individually.

 © The Author(s)
